# Mediating Role of Green Supply Chain Management Between Lean Manufacturing Practices and Sustainable Performance

**DOI:** 10.3389/fpsyg.2021.810504

**Published:** 2022-01-03

**Authors:** Fazal Hussain Awan, Liu Dunnan, Khalid Jamil, Sohaib Mustafa, Muhammad Atif, Rana Faizan Gul, Qin Guangyu

**Affiliations:** ^1^School of Economics and Management, North China Electric Power University, Beijing, China; ^2^Beijing Key Laboratory of New Energy and Low-Carbon Development, North China Electric Power University, Beijing, China; ^3^College of Economics and Management, Beijing University of Technology, Beijing, China; ^4^Department of Sociology, Government College University Faisalabad, Faisalabad, Pakistan

**Keywords:** lean practices, green supply chain management, sustainable performance, social sustainability, Pakistan

## Abstract

Manufacturing companies in today's industrial world are seeking to use the new manufacturing process methods. The primary goal of corporations is to achieve optimum production while deploying minimal capital. The fundamental purpose of this study is to examine the influence of various lean manufacturing practices on the sustainability performance of companies and the mediating role of green supply chain management (GSCM). The data was gathered using questionnaires from 250 Pakistani manufacturing firms and analyzed using AMOS 25. Results demonstrate that process and equipment, product design, supplier relationships, and customer relationships significantly affect sustainable performance. It is also recognized that Green Supply Chain Management mediates the interaction between HR processes, product design, supplier relationship, customer relationship, and environmental performance. The findings of this study will enable managers and decision-makers of manufacturing companies to increase sustainable efficiency and reduce waste through the use of lean manufacturing and GSCM implementation.

## Introduction

Many characteristics describe the management of the supply chain by various parameters. The parameters include intense competition, an increased requirement for cleaner products, environmental maintenance problems, and the pressure to decrease and handle waste problems. Customers' primary demand is to use the “cleaner” products to reduce waste, environmental decline, and problems related to contamination and pollution (Henao et al., 2019). “Vision for environmental sustainability” type-projects are required for maintaining a difficult advantage in the supply chain department. The main aim of prominent and successful businesses is to combine the two dominant strategies of “lean” and “green” for the extraction of waste and scrap produced as a part of routine operational processes. Notable cost savings for supply chain functions may be significant by merging lean and green thinking to identify, reduce, and remove the excess usage of resources and the production waste (Iranmanesh et al., 2019).

Lean manufacturing is defined as “a procedure always designed to lower the costs and waste products in manufacturing firms.” Huo et al. (2019) say that lean manufacturing, in their words, is “a business method and procedure that can improve the business performance for customers' satisfaction and improve the line.” Research has proven that lean manufacturing has prominent effects on the firms' manufacturing and operation. There are different central practices present, which are the factors for the formation of lean manufacturing (Hao et al., 2021). Researchers are working on lean manufacturing and are keen to find other best ways for lean manufacturing. They have recommended different lean practices for industries such as those surrounding electronics and the automotive sector. Rathore et al. (2020) and many other researchers identified many main areas for lean production, and they suggested that these lean practices can be implemented in different manufacturing sectors.

Different research types have revealed the interconnection between lean manufacturing and environmental performance and performance related to finance. These researches have two main restrictions (Huo et al., 2019). First, it is noticed that in past literature, there is less focus on the impact of lean manufacturing practices on the sustainability performance of the firm. To work within a competitive environment and keep balance in the working environment, manufacturing organizations should have equilibrium in their environmental, economic, and social performance (Chavez et al., 2020). The manufactures must have the capability to learn that lean practices include noticeable positive impacts on different parameters of maintenance and not only a single parameter. Secondly, most of the research done previously has conducted a test that has the effect of overall lean manufacturing on organization performance (Kovilage, 2021). The main picture that shows the impact on firm performance is indicated by the interconnection between sustainable performance and these lean manufacturing practices with multiple branches in different areas of the firm (Habidin et al., 2013).

In recent years, the fast industrial refurbishment has made its way to have negative impacts related to environmental issues, including greenhouse gas emissions, poisonous pollution, and chemical overflow (Henao et al., 2019). In acknowledging the growing global environmental awareness, GSCM has made itself clear as a point that takes the maintainable elements and combined with the environmental issues for the upstream and downstream supply chain to have intra-and inter-firm management (Hartini and Ciptomulyono, 2015). To reduce the environmental waste in organizations and improve ecological performance, the green prototype intentionally decreases environmental issues and disputable ecological impacts. Researchers and practitioners are focusing on and giving their intentions on environmentally conscious business practices. Iranmanesh et al. (2019) showed the lean scheme's effect on all the phases of a product's life series. By raising the ecological efficiency and decreasing the environmental insecurity, issues, and impact, lean scheme on the performance of GSCM has risen to the surface as a new and prominent prototype for projects to achieve the objectives of profitable yields and market share (Pearce et al., 2018).

This paper has a contribution to the literature and research in two possible points. Firstly, a mindful and contextual structure was disclosed, connecting the six lean practices and sustainable performance (environmental, financial, and social) of manufacturing firms. Secondly, we logically explain or assess the mediating role of GSCM between the connection of lean manufacturing and sustainable performance. There is no mediating role defined in the literature. Therefore, it is compulsory to renew the rational understanding of the relationship between lean manufacturing practices and sustainable performance and GSCM. This paper also has a remarkable contribution for managers. It can guide managers to adopt the best lean approach to increase and improve the sustainable performance of their firm by implementing GSCM.

## Literature Review

### Lean Manufacturing Practices and Sustainable Performance

Kamble et al. (2020) used value stream mapping (VSM) to give evidence of the development of lean (waste elimination) manufacturing. The research of the authors compares the current state and future roadmaps for knowledge about the advantages of lean manufacturing, and their research has clearly shown and predicted the value of steam metrics.

Another parameter of maintenance and sustainability is the interconnection between lean manufacturing and financial performance as discussed according to the previous research. Orji and Liu (2020) described a more significant contribution to the financial returns. There will be more cost reduction through waste reduction because of the endowment to the environment by lean manufacturing. According to Hussain et al. (2019), lean manufacturing also provides information on the importance of pollution reduction and contributes to financial performance besides reducing waste management costs. There is a positive response to lean manufacturing and financial performance in the research article (Burawat, 2019).

Social performance is another perspective of maintenance, and besides the requirement of lean manufacturing, there is also demand for social performance by customers for improving the financial and environmental results (Sajan et al., 2017). Research must be done on social responsibility along with lean manufacturing to emphasize their importance. In some literature, there is a concept about lean manufacturing that reduces stress, increases responsible autonomy, and leads to some inborn motivation. It also leads to some passionate work (Ben Ruben et al., 2019).

### Green Supply Chain Management

GSCM is research mainly aimed to merge the environmental requirements into supply chain management options. This was the first and fundamental proposal given by Michigan State University in 1996. Bai et al. (2019) wanted to combine the lean manipulation efforts with financial performance as identified by awards and stock market returns. Lean management initiatives described the positive and negative results for market gains and the cost savings. There are some mixed results, as shown by lean management and financial performances. There are some identical facts described according to Vinodh et al. (2016) as matching the above-described results. There are some positively associated market reactions for the munificent environmental gifts. There are also negative impacts for voluntary emissions depletion, and ISO 14001 certifications are also related to positive market reactions (Helleno et al., 2017). This research type is related to the markets having different responses for different categories of lean inventiveness. Vanichchinchai (2019) suggested that the implementation of lean inventiveness could remove or lessen unseen waste and unproductive operations. They strengthen the overlaps and regularity with the management programs' policies and technologies, skeletal systems, and other programs related to qualitative management.

Hartini and Ciptomulyono (2015) claimed that there are five different parameters of GSCM, including other strategic plans like lean tasks, lean format, lean production, reverse logistics, and waste manipulation plans. This literature is concerned with the issues and has a short introduction about the management plans related to the supply chain field and a brief description of the GSCM discipline, lean designs, and lean operations. The paper also emphasizes the importance of lean processes for the motives of the organization. Through their research, Vinodh et al. (2016) depicted that green and lean principles have a significant contribution toward pollution control. Implementing both projects in the same period can contribute more toward the performance of operations than implementing the two sequentially. They can solve issues and have restrictions on practices. The research of Huo et al. (2019) is based on the manufacturing sector of Asian Emerging Economics (AEEs) to study the interconnection between GSCM practices and organizational performance. The findings revealed the fact that apart from the better understanding of the main three parameters: economic, environmental, and social areas, the GSCM practices-performance relationships are greatly moderated by industrial facts such that their type, organization structure and size, certification related to ISO, and the export orientation. This main aim of this literature is to step in and start updating the academic field by acquiring the meta-analysis mode for confirming the GSCM practice presentation relationships in the production sector of AEE as the meta-analysis technique is not yet applied in the management of the supply chain. Moreover, this research study also motivates managers and policymakers to adopt GSCM practices for improving firm performance (Singh et al., 2020).

Haiyun et al. (2021) suggested that lean practice contributes to combating global warming as it is an asset in decreasing the international factors of production, i.e., pollution. Much research and study results have proved that lean operations and transport have successfully reduced pollution and increased efficiency. This primary environmental concern is to limit the excesses of carbon dioxide emission, and these lean practices have made it possible through higher usage of resources in a short period. Bhatia and Gangwani (2021) proposed an idea to estimate and judge the progress or development of businesses in terms of lean implementation. For this estimation, ~5 different organizations were considered for the case study related to the verification of the conceptual framework. This conduction proved that the initiatives taken for the conduction are appropriate for the lean and green framework. These results were of higher scores, which proved a good interconnection between green production and lean production (Stekelorum et al., 2021).

### Relationship Between Process and Equipment and Sustainable Performance

Some representatives have the intention of developing a regular and streamlined flow for the manufacturing process. These are the processes and equipment which show improvement capabilities, such as the usage of equipment that is “error-proof,” cycle time reduction, availability, and the assurance of machines (Mathiyazhagan et al., 2021). Waste material and defects are developed as a result of incorrect processing and over-processing. False processing and over-processing in machinery result in high-risk materials, an increase in water consumption, and the depletion of energy. According to Tripathi et al. (2021), that the above-mentioned proves and equipment have a better effect as they have the focal points related to the development of a better environment and also an asset in the identification of spills and leaks and help to decrease the defects and energy usage by depletion of pollution. Many benefits have been observed as a result of the implementation of process and equipment practices (Chavez et al., 2020). The asset is the lack of water and energy usage. Along with that, they have better effects on the activities of the firm, which leads to the improvement in the environment of the firm and helps provide a healthy environment. Therefore, it is said according to the hypothesis that:

H1: Process and Equipment have a significant impact on Sustainable Performance.

### Relationship Between Planning and Control and Sustainable Performance

Scheduling strategies are linked with the planning and control practices in lean manufacturing to manufacture and coordinate market demand. There are many factors that are assets to the achievement of a higher production rate (Sakthi Nagaraj and Jeyapaul, 2020). The main areas of waste are the scheduling and depletions in the initial materials and workforce usage, which are the critical concern of lean planning and control. The primary concern of effective schedules gives effective results. The smoother flow of production, which is obtained as the elimination of imbalances in the production line, is also the best result of small lot sizing (Iqbal et al., 2020). The depletion of waste from overproduction and the reduction in storage is also possible as the consequences of the methods such as Kanban and lot size depletion. The delivery time is not being affected by the implementation of lean and control practices. They help in the diminution of materials and components that are part of the manufacturing process. Jermsittiparsert et al. (2020) also suggested that the pull advancement significantly reduces waste caused by the damaged products and minimizes the work process and floor space utilization. Hence, the above-mentioned statement that the implementation could minimize process waste and fault material of planning and control practices. Accordingly, the following hypothesis is posited:

H2: Planning and Control has a significant impact on Sustainable Performance.

### Relationship Between HR Practice and Sustainable Performance

The significant development of the proper work atmosphere and human capital evolution results from HR practices' best implementation (Rathore et al., 2020). The lean objectives are employees' authority, involvement, autonomous problem solving, self-directed work teams, problem-solving groups, and formal training plans. HR is the basis of quality enhancement programs that are founded as the result of lean manufacturing implementation. Kumar et al. (2020) showed that HR has a prominent effect on pollution and waste depletion. If the organization has trained and self-disciplined employees, it can have better usage of resources and efficient waste reduction. An organization's financial performance can be improved by providing the employees the authority to take action for the reduction of waste and pollution, which is only applicable by the implementation of HR. This leads to the following hypothesis:

H3: HR Practices has a significant impact on Sustainable Performance.

### Relationship Between Product Design and Sustainable Performance

Multifunctional pattern teams, design for production, product modularization, and parts of some high merit are the item designs implemented in lean manufacturing (Jamil et al., 2021a). This product design's primary concern is to provide easy access for the manufacturing of product maintenance by decreasing the material usage that results in the smooth and linear manufacturing process and makes the best use of firms' resources (Sahu et al., 2020). This product design is also an asset in depressing power usage by making product design with a joined-up part of the current production process. For the best and most effective achievement of this, all waste materials must be extracted. Buer et al. (2021) invented a positive connection between the design format and firms; economic results. This leads to the following hypothesis:

H4: Product Design has a significant impact on Sustainable Performance.

### Relationship Between Supplier Relationship and Sustainable Performance

Supplier Relationship is concerned with the dealings of suppliers regarding several tasks such as concerns about quality, ensuring just-in-time delivery, long term interconnection, and supplier concern about the product format and evolution proves to respond to the suppliers' performance (Naseem et al., 2020). There is importance for implementing supplier relationship practices such as face-to-face decisions and transmission related to the production, which is the primary concern of firms for solving the supplier performance issues. If these issues are solved, then this is an asset to achieve a certain level of performance (Mellado and Lou, 2020). Best environmental, social, and financial performance is possible by developing innovative technologies, which can be possible by developing relationships with the supplier. The development of productivity and environmental performance is also possible through the implementation of supplier relationships. Less energy usage, fewer defects, and waste reduction are possible if there are supplier interconnection best practices, as mentioned by Yadav et al. (2020). This results in better and more compatible enhancement. Closer interconnection with suppliers provides a chance to increase the suppliers' accomplishments, which can assist in developing the best environmental performance through innovative practices and materials. Therefore, it is hypothesized that

H5: Supplier Relationship practices have a significant impact on Sustainable Performance.

### Relationship Between Customer Relationship and Sustainable Performance

The firm aims to develop long-term relationships with the customers to increase customer satisfaction and manage their complaints, and this is applicable by the implementation of effective customer relationships (Gul et al., 2021). What are the needs of the customer? And how the loyalty products, services, and the process can be provided? The answer to these issues is possible through a close relationship with the customers involving their opinions on product design and other information related to the output of the firm. Loyalty products and services mark a differentiation from other providers and thus also add value. Customers play an important and efficient role in developing innovative manufacturing systems, which led to the development of the lean manufacturing concept (Jamil et al., 2021b). Good customer relationships lead to the importance of the dissemination of environmental and social manufacturing practices that can improve social and ecological performance. The importance of social and environmental practices strategies is the need for higher accounts and is highly recommended by the customers. A closer relationship between the suppliers and customers is possible and accessible by providing clear production, as indicated by Mohsin et al. (2021). The enhancement and improvement in the social, environmental, and financial performance are possible by the interlinkage of suppliers, workers, and customers for the study, development, and partnership. As such, the following hypothesis is developed:

H6: Customer Relationship has a significant impact on Sustainable Performance.

### The Mediating Role of Green Supply Chain Management

As a result of lean manufacturing and more efficient procedures, there has been a significant decrease in emissions from transportation and production activities. Carbon dioxide is a greenhouse gas, which contributes to climate change and is a worldwide issue. Reducing the amount of carbon dioxide released into the atmosphere is one way to combat global warming and pollution (Jamil et al., 2021b). As a consequence of reducing waste, lean manufacturing may assist in reducing pollution since it reduces cycle times and increases resource utilization. To evaluate firms in terms of their supply chain implementation, Sarfraz et al. (2021) suggested an evaluation system. The conceptual framework was validated by conducting a multiple case study of five distinct manufacturing companies. The research found that organizations with high ratings have a solid relationship between green and lean implementation. According to the findings, the conceptual framework's green and lean assessment framework is well-represented by its chosen projects.

**Figure 1:**
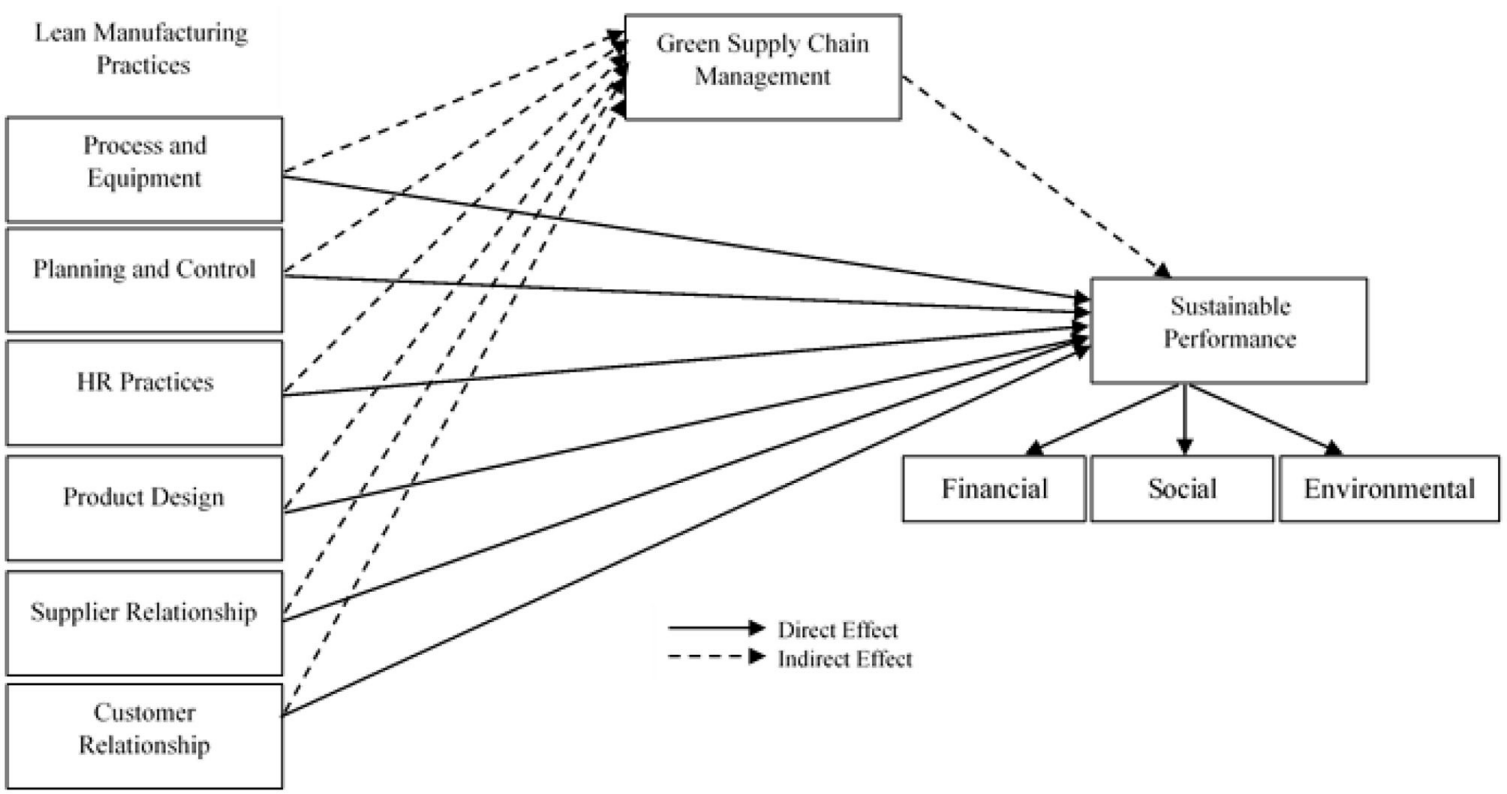
Conceptual framework.

The main achievement and the goal of lean management are to provide innovative products and services useful in quality and lesser in cost. According to this lean context, waste is described as “something other than the required amount of equipment, stuff like materials, parts, resources, and time (Dunnan et al., 2020). These are important parameters for adding value to the product.” Transport, inventory, motion, waiting, over-processing, and overproduction are waste parameters that are included in the forms of waste. These are all non- value-adding operations that impact quality and performance, but customers are not concerned with such problems. Organizations working on lean practices have implemented these practices in different sectors to improve efficiency and competitiveness (Viles et al., 2021). The GSCM has many benefits, and there is proof in this literature regarding the benefits of lean practices. The benefits of these lean practices can be derived by spreading lean practices through lean practices. According to Ikumapayi et al. (2020), sustainability is the next productivity stage of lean management to encourage and motivate external waste to decrease the GSCM and improve social conditions globally. Lean practice can be implemented in the industries and across the supply chain, aiming for orderly distribution and delivery (Tiwari et al., 2020). This can eliminate waste, and improve the quality and customer service at all stages of the supply chain. Hence, we proposed the following hypotheses (see Figure 1 for all relationships).

H7: GSCM significantly mediates the relationship of (a) Process equipment, (b) Manufacturing Planning, (c) Product Design, (d) Supplier Relationship, and (e) Customer Relationship on Sustainable Performance.

## Methods

### Data Collection and Sample Size

The study's sampling frame includes all manufacturing units in Pakistan that have implemented lean practices within the production process. A well-structured questionnaire was delivered to the general manager, production managers, and the senior manager directly involved with the manufacturing process. They have the best knowledge and experience to respond to lean practices. As lean is a multidimensional approach, different departmental managers such as the HR department, customer care department, supply chain, logistics, and engineering department were involved in filling the questionnaire. The list of manufacturing units was obtained from the Securities and Exchange Commission of Pakistan (SECP). By keeping in view the sample size, the study used a well-reputed and globally implemented sample size formula focusing on a finite population introduced by Krejcie and Morgan (1970). Furthermore, to improve the generalizability of the findings, questionnaires were delivered to 350 targeted respondents in different manufacturing firms, from which 250 complete and usable questionnaires were received back with a response rate of 71%.

Descriptive analysis shows that 40% of the respondents were from the textile sector, 15% were pharmaceutical, 15% were from food processing, 10% were from the chemical sector, 10% were from the automotive sector, and 10 % were from others.

### Questionnaire and Measurements

A comprehensive literature review was conducted to ascertain the items observed regarding the evaluation of the relationship between latent variables. The questionnaire was developed and comprised of 72 questions in eight parts by adopting items from different studies. On a Likert scale from one to five on all item scales, respondents had to assess their viewpoint.

Lean Practices were assessed with items taken from the studies of Panizzolo (1998) on a five-point Likert scale of one (not at all) to five (Very High). The scale was made up of thirty items. Furthermore, GSCM was evaluated with items taken from the study of Singh et al. (2020) on a five-point Likert scale of one (not at all) to five (Very High). The scale was composed of 25 items. Also, sustainable performance (Environmental performance, social performance, and financial performance) was evaluated on a five-point Likert scale, one (strongly disagree) to five (strongly agree) with 17 items adopted from Zailani et al. (2015). See Appendix A for questionnaire items.

## Results

In AMOS 25.0, we ran structural equation modeling (SEM) to validate the proposed measurement and structural model of the study. The reason for running AMOS SEM is that AMOS is a powerful tool for data analysis that performs factor analysis and regression analysis simultaneously (Sarstedt et al., 2014). First, we conducted confirmatory factor analysis (CFA) in AMOS 25.0 to confirm the quality of fit indices of the proposed measurement model. To do so, 10 variables, including (Process and Equipment, Manufacturing Planning and Control, Product Design, Supplier Relationship, GSCM, Customer Relationship, HR Practices, and Second-Order Sustainable Performance (Environmental Performance, Financial Performance, Social Performance)were interlinked and analyzed using CFA. The CFA results indicated that the proposed measurement model is an acceptable, excellent fit and achieved the model fitness cut-off values shown by Hair et al. (2014). For the present study, the CFA results confirm the model fit indices (see Table 1).

**Table 1:** Model fit indices.

**Fitness indices**	**Measure**	**Measurement model**	**Structural model**	**Threshold values**
Chi-square/df (CMIN/DF)	CMIN/DF	2.345	1.45	<3.00
Comparative fit index	CFI	0.939	0.999	>0.95
Standardized root mean square residual	SRMR	0.078	0.071	<0.08
Root mean-square error of approximation	RMSEA	0.068	0.061	<0.06
*P*-value	P-Close	0.05	0.058	>0.05

### Reliability and Validity of the Constructs

We also adopted the guidelines for evaluating scale reliability and validity by Hair et al. (2014) and Fornell and Larcker (1981). We tested Cronbach's alpha (CA), composite reliability (CR), average extracted variance (AVE), and items loading (IL) for reliability and validity analysis. The results demonstrate that all variables achieved the threshold values for reliability and validity. The values of IL (0.524–0.930), CA (0.822–0.963), CR (0.822–0.962), and AVE (0.569–0.846) are greater than the respective cut-off threshold, which demonstrates that all measures are valid (see Table 2). Besides, the Fornell and Larcker (1981) method was used to test the discriminatory validity of the scales. The findings revealed that for each construct, the square root for AVE is higher than the values of intercorrelations of the study variables, illustrating the scales' excellent discriminant validity.

**Table 2:** Properties of measurement model.

**Latent** **constructs**	**Items Range**	**CA**	**CR**	**AVE**	**MSV**	**MaxR(H)**
PAE	0.696–0.801	0.840	0.841	0.569	0.029	0.845
MPC	0.771–0.834	0.822	0.822	0.606	0.119	0.830
PD	0.726–0.847	0.842	0.844	0.575	0.562	0.855
HRP	0.737–0.812	0.902	0.903	0.607	0.524	0.904
CR	0.815–0.885	0.803	0.890	0.731	0.119	0.895
GSCM	0.595–0.846	0.963	0.962	0.565	0.562	0.967
SR	0.770–0.922	0.880	0.879	0.709	0.031	0.903
SAP	0.524–0.930	0.956	0.943	0.846	0.494	0.957

In AMOS, the validity of the instruments is measured in two ways. We determined the measurement model by testing the convergent validity by Hair et al. (2016) looking at the values of average variance obtained (>0.5) and composite reliability (>0.7). As shown in Table 2, all the deals fulfilled the suggested threshold, and therefore, convergent validity was verified.

Table 3 displays the discriminant validity assessment whereby the HTMT ratios were all below the 0.85 cut-off value. The confidence intervals do not include a zero or one, as suggested by. Thus, we can conclude that (Sarstedt et al., 2014) the measures used in this are reliable, valid, and distinct. The values that lie in off-diagonal are smaller than the average variance's square root (highlighted on the diagonal), supporting the scales' satisfactory discriminant validity.

**Table 3:** Discriminant validity.

	**PAE**	**MPC**	**PD**	**HRP**	**CR**	**GSCM**	**SR**	**SUS**
PAE								
MPC	0.073							
PD	0.000	0.126						
HRP	0.011	0.213	0.69					
CR	0.172	0.358	0.044	0.111				
GSCM	0.003	0.112	0.758	0.739	0.043			
SR	0.004	0.088	0.205	0.160	0.059	0.148		
SUS	0.036	0.090	0.680	0.703	0.065	0.793	0.162	

### Hypotheses Testing Results

The Confirmatory factor analysis results for the structural model revealed that the model is a good fit and achieved the cut-off values of model fitness, recommended by Hair et al. (2014). The Confirmatory factor analysis findings confirm the goodness of model fit indices meet the criteria (see Table 1). In the next step, the standardized path values were estimated after the confirmation of goodness model fit indices of the structural model using the maximum likelihood method in AMOS 25. First, we assessed the direct relationships before looking at the mediation effects. The results reveal that PAE was positively related to sustainable performance (*β* = 0.144, *p* < 0.01) but MPC (*β* = −0.013, *p* = 0.803) and HRP (*β* = 0.025, *p* = 0.800) were not significantly related to sustainable performance, which supports H1. In contrast, H2 and H3 are not supported. Furthermore, for H4, H5, and H6: PD (*β* = 0.153, *p* = 0.031), SR (*β* = 0.216, *p* < 0.01), and CR (*β* = 0.265, *p* < 0.01) were also positively related to sustainable performance giving support for H4, H5, and H6 of our study. Also, we have two endogenous constructs in our model GSCM and sustainable performance. The *R*^2^ for GSCM was 0.640 (*Q*^2^ = 0.254), and sustainable performance was 0.719 (*Q*^2^ = 374), which indicates that their predictors can explain 64, 69.2, and 0.71.9% of the variance in the respective constructs. *Q*^2^ values >0 indicate sufficient predictive relevance.

Similarly, to assess the mediating relations, six mediating relationships were proposed. These linkages were proposed to check the mediating effects of GSCM in the relationships among the dimensions of lean practices and sustainable performance. The estimated results from mediation testing are presented in Table 4. To test the mediation effect, we used the bootstrapping the indirect effect method (Preacher and Hayes, 2008) with a 5,000 resample and validate the mediation hypotheses 7a to 7f. The indirect effect of PAE → GSCM → sustainable performance (*β* = −0.009, *p* = 0.525, BCI LL = −0.043 & BCI UL = 0.020) and MPC → GSCM → sustainable performance (β = −0.006, *p* = 0.505, BCI LL = −0.029& BCI UL = 0.021) indicating the indirect effect is statistically insignificant. which do not give support for H7a and H7b of this study. Furthermore, for H7c, H7d, H7e, and H7f, the results reveal that GSCM significantly mediates the relationship between HRP and sustainable performance (β=0.065, p = 0.019), PD and sustainable performance (β=0.094, p < 0.01), SR and sustainable performance (β=0.085, p < 0.01) and, CR and sustainable performance (β=0.050, p < 0.01), indicating the indirect effects are statistically significant at the 0.01 level. This supports H7c, H7d, H7e, and H7f of this study (see Table 4).

**Table 4:** Hypotheses testing results.

		**Estimates**	**Std error**	**BCI LL**	**BCI UL**	***P*** **-values**	**Decision**
*H1*	PAE -> SAP	0.144	0.055	0.038	0.245	0.007	Accepted
*H2*	MPC -> SAP	−0.013	0.052	−0.115	0.092	0.803	Rejected
*H3*	HRP -> SAP	0.025	0.072	−0.121	0.163	0.800	Rejected
*H4*	PD -> SAP	0.153	0.070	0.005	0.277	0.031	Accepted
*H5*	SR -> SAP	0.216	0.070	0.081	0.345	0.001	Accepted
*H6*	CR -> SAP	0.265	0.082	0.095	0.420	0.002	Accepted
*H7a*	PAE -> GSCM -> SAP	−0.009	0.017	−0.043	0.020	0.525	Rejected
*H7b*	MPC -> GSCM -> SAP	−0.006	0.013	−0.029	0.021	0.505	Rejected
*H7c*	HRP -> GSCM -> SAP	0.065	0.028	0.022	0.127	0.019	Accepted
*H7d*	PD -> GSCM -> SAP	0.094	0.032	0.037	0.164	0.003	Accepted
*H7e*	SR -> GSCM -> SAP	0.085	0.030	0.033	0.149	0.005	Accepted
*H7f*	CR -> GSCM -> SAP	0.050	0.019	0.019	0.091	0.004	Accepted
*Endogenous Constructs*	*GSCM*	*SAP*					
*R^2^*	0.640	0.719					
*Q^2^*	0.254	0.374					

*PAE, Process and Equipment; MPC, Manufacturing Planning and Control; HRP, HR Practices; PD, Product Design; SR, Supplier Relationship; CR, Customer Relationship; SAP, Sustainable Performance; GSCM, Green Supply Chain Management*.

## Discussion

The findings of the current study are in line with the investigations of Panizzolo (1998) and Dieste et al. (2020), who proved that there is a dominant impact of over-processing and the usage of old machinery also give out the depletion of energy and resources and also enhance the production of emissions. To create a uniform and streamlined flow in the production process, some practices are required. Those practices include cycle time depletion, decreasing set-up times, order and cleaning, and the usage of “error-proof” methods. The environmental advantages of lean manufacturing and production can be increased by implementing the value stream mapping through fewer problems, lower energy usage, and less wear. This proposal furnishes with the procedure for better development, cleaning, and categorizing the work environment, which moves to the fast authorization of system problems such as leaks and depletion in the chemicals and materials used (Sakthi Nagaraj and Jeyapaul, 2020). Furthermore, manufacturing firms can improve their performance by lowering their energy usage and implementing the lean process and equipment practices.

The above results of this study have made the point clear that planning and control practices have no prominent effect on sustainable performance, especially in the textile and chemical sectors because these two require more planning and control practices. This may be why there are more negative aspects to the market than positive aspects because the chemical sector requires a more controlled environment. According to the study of Mojumder and Singh (2021), the frequent changeover requires more usage of material and the high pressure on employees for lot size reduction in manufacturing and control practice. According to Haiyun et al. (2021), the side issues of planning and control practices impact the environment more than the benefits such as Kanban and visual control.

The interconnection between HR practices and sustainable performance was not proved in this study as in the chemical and food sectors there is necessarily more focus on the latest technology irrespective of human resource participation. This result does not follow the findings of Vanichchinchai (2019), which lead to the concept that HR practices can lead to lower contamination and waste production, especially in the textile sector. The team leadership and the importance of workers for preventing pollution are essential concepts provided by Ghosh (2013). According to his research, team advancement—comprising engineers, managers, and production staff—can play an important role in reducing scrap and improving environmental results. Hussain et al. (2019) contributed the evidence supporting the importance of HR agreement in his research, having found that involving the employees and granting them the best educational training can lead to a better environmental approach. Trained and experienced staff learn better and have a better capacity to solve problems that can prevent pollution and reduce materials usage. Hence both positive and negative aspects are present, which can show that there is no essential relation between HR practice and sustainable performance.

The above results have shown a positive relationship between product design and sustainable performance. The product design aims to eliminate unnecessary steps and increase the simplification of the process. Product design practices can contribute to resource usage and increase the level of supercity by decreasing or eliminating the waste from the production process. This can have a better effect on the work pressure. The main focus is to reduce waste by adequately implementing the product design practices (Pearce et al., 2018). Organizations that want better performance and wanted to minimize waste must implement the product design practices.

According to the above results, the other factor that positively impacts sustainable performance is supplier relationship practices. According to Samad et al. (2021), supply maintenance practices can extract the waste and increase inventory usage by depleting the variability in supply. There is a need to have a close working relationship with the suppliers for the early stage of development and increase process quality and waste reduction. The exchange of information and communication with the suppliers can increase the supplier's ability to learn about the requirements needed for the best products in the industry.

The above results have also proved a positive impact on customer relationships on sustainable performance. The adoption of customer relationships in the organization can lead to the implementation of the best manufacturing practices, according to Helleno et al. (2017). For a better image of the organization in society's eyes and to have the best relationship with the customers, the implementation of customer relationships is efficiently needed in the organization. To satisfy the customers and increase the level of satisfaction, there is a need to meet the customer's social and environmental demands.

As discussed earlier by Singh et al. (2020), if a firm wants to get significant results from lean practices, then it should adopt GSCM, which helps to get sustainable performance. So, in the present study, GSCM proposed a mediator to increase lean practices, leading to enhanced sustainability performance (Kamble et al., 2020). This study shows that GSCM mediates the relationship between HR practices, product design, supplier relationships, customer relationships, and sustainable performance. The results express that lean practices such as HR practices, product design, supplier relationship, and customer relationship significantly impact sustainable performance if a firm uses GSCM and the reason of having no mediation role of GSCM between process and equipment, manufacturing planning and control, and sustainable performance that the concept of lean and GSCM within Pakistani manufacturing firms is at an initial stage. According to Mojumder and Singh (2021), lean practices take a long time to take part in the firm's sustainable performance.

The primary demand of this research was to indicate the interconnection between lean manufacturing practices and the sustainable performance of the production companies of Pakistan by having a GSCM as a mediator. The above results have proved that product design, supplier design, and customer relationships have tremendous impacts on sustainable performance. The positive effects of GSCM on sustainable performance are because of the impact of process and supplier relationships (Stekelorum et al., 2021).

Working toward GSCM as a potential tool to improve the sustainability of organizations, the results showed a small incremental difference but a considerable improvement in organizations' sustainability indicators (Orji and Liu, 2020). To sustain a business, it is necessary to properly utilize human resources, produce better designed products, and improve customer relations. Different aspects of the lean and green strategies have been studied as they can be used to support organizational sustainability (Kovilage, 2021). The study highlights the significance of the GSCM in organizational sustainability through environmental performance, economic performance, and social performance in Pakistan. Thinking Lean can increase exceptional performance in the organization only when a negligent focus is placed on waste identification and elimination.

## Practical Implications

The findings of this study provided the best endowment for managers. These findings will enable the managers and decision-makers of manufacturing companies to increase sustainable efficiency and reduce waste through the use of lean manufacturing and GSCM implementation. The managers have to understand different terms related to the progress of the organization. This can help the managers take a step to add a significant contribution toward the progress of the firm by implementing the manufacturing model practices. There are positive effects of process and equipment, product design, and supplier relationship on the sustainable performance of the company; hence, these practices must be implemented by managers for their positive effect. To increase the impact of process and equipment, there must be the implementation of GSCM development by managers of the organization. This literature has emphasized lean manufacturing practices from the research view and has highlighted the interconnection between GSCM and sustainable performance.

Readers will come to know the practical significance of small incremental improvements and innovative activities that consider green supply chain management and that relate to better manufacturing and service turns into a mediator. Besides, the significance of lean and green strategies in real manufacturing environments can be relatively well-studied, as the present studies suggest.

## Limitations and Future Research

Although the study has achieved its objectives, certain limitations need to be considered to generalize its findings. First of all, the study's nature is cross-sectional, which cannot show the dynamic nature of sustainable performance. There is a need for a longitudinal study that can present a clear picture of lean manufacturing practices and their impact on sustainable performance. The samples were also limited to Pakistan only, and there is a need to implement this model in other countries before it can be generalized.

## Data Availability Statement

The raw data supporting the conclusions of this article will be made available by the authors, without undue reservation.

## Author Contributions

All authors listed have made a substantial, direct, and intellectual contribution to the work and approved it for publication.

## Funding

This work was partly supported by National Social Science Foundation of China (No. 19ZDA081).

## Conflict of Interest

The authors declare that the research was conducted in the absence of any commercial or financial relationships that could be construed as a potential conflict of interest.

## Publisher's Note

All claims expressed in this article are solely those of the authors and do not necessarily represent those of their affiliated organizations, or those of the publisher, the editors and the reviewers. Any product that may be evaluated in this article, or claim that may be made by its manufacturer, is not guaranteed or endorsed by the publisher.
