# Supplementary material for: Mediating Role of Green Supply Chain Management Between Lean Manufacturing Practices and Sustainable Performance
Source: Front Psychol. 2022 Jan 3;12:810504. doi: 10.3389/fpsyg.2021.810504 (PMC8761733; doi:10.3389/fpsyg.2021.810504)
Supplement: Supplementary file 1 [file Data_Sheet_1.docx]

**Appendix A: Questionnaire**

**Scale**

| **Not at all** | **Low Extent** | **Moderate Extend** | | **High Extent** | **Very High** |
| --- | --- | --- | --- | --- | --- |
| NA | LE | | ME | HE | VH |

**Process and Equipment**

| My firm does implement……………. | NA | LE | ME | HE | VH |
| --- | --- | --- | --- | --- | --- |
| Set up reduction | 1 | 2 | 3 | 4 | 5 |
| Continuous flow of production | 1 | 2 | 3 | 4 | 5 |
| Order and cleanliness (5S) | 1 | 2 | 3 | 4 | 5 |
| Cycle time reduction | 1 | 2 | 3 | 4 | 5 |
| Value stream mapping | 1 | 2 | 3 | 4 | 5 |
| error proofing techniques/ Pokayoke | 1 | 2 | 3 | 4 | 5 |

**Manufacturing Planning and Control**

| My firm does implement……………. | NA | LE | ME | HE | VH |
| --- | --- | --- | --- | --- | --- |
| Pull system/Kanban | 1 | 2 | 3 | 4 | 5 |
| Planning and scheduling strategies | 1 | 2 | 3 | 4 | 5 |
| Lot size reduction | 1 | 2 | 3 | 4 | 5 |
| Visual control of shop floor | 1 | 2 | 3 | 4 | 5 |

**Product Design**

|  | NA | LE | ME | HE | VH |
| --- | --- | --- | --- | --- | --- |
| My firm do implement parts standardization | 1 | 2 | 3 | 4 | 5 |
| My firm do implement product modularization | 1 | 2 | 3 | 4 | 5 |
| My firm do implement design for manufacturability | 1 | 2 | 3 | 4 | 5 |
| My firm has multifunctional design teams | 1 | 2 | 3 | 4 | 5 |

**Human Resource Practices**

|  | NA | LE | ME | HE | VH |
| --- | --- | --- | --- | --- | --- |
| My firm has multifunctional (multi skill) workers | 1 | 2 | 3 | 4 | 5 |
| My firm gives workers a broader range of tasks. | 1 | 2 | 3 | 4 | 5 |
| In my firm, workers undergo cross functional training | 1 | 2 | 3 | 4 | 5 |
| In my firm we have expansion of autonomy and responsibility | 1 | 2 | 3 | 4 | 5 |
| In my firm, workers involve in continuous improvement efforts | 1 | 2 | 3 | 4 | 5 |
| In my firm, shop floor employees are key to problem solving teams | 1 | 2 | 3 | 4 | 5 |
| In my firm, team members opinion and ideas (suggestions) are considered before making decisions | 1 | 2 | 3 | 4 | 5 |

**Supplier Relationships**

|  | NA | LE | ME | HE | VH |
| --- | --- | --- | --- | --- | --- |
| We strive to establish the long-term relationship with our suppliers | 1 | 2 | 3 | 4 | 5 |
| Our key suppliers deliver to plant on the just-in-time (JIT) basis | 1 | 2 | 3 | 4 | 5 |
| Suppliers are directly involved in the new product  development process | 1 | 2 | 3 | 4 | 5 |
| Our key suppliers are located in close proximity to our plant | 1 | 2 | 3 | 4 | 5 |
| We evaluate suppliers on the basis of total cost and not per unit price | 1 | 2 | 3 | 4 | 5 |

**Customer Relationships**

|  | NA | LE | ME | HE | VH |
| --- | --- | --- | --- | --- | --- |
| We are in close contact with our customers | 1 | 2 | 3 | 4 | 5 |
| Our customers are actively involved in product design development | 1 | 2 | 3 | 4 | 5 |
| Our customers frequently share current and future demand  information with marketing department | 1 | 2 | 3 | 4 | 5 |
| Our customers frequently give us feedback on quality and  delivery performance | 1 | 2 | 3 | 4 | 5 |

**Green Supply Chain Management**

|  | NA | LE | ME | HE | VH |
| --- | --- | --- | --- | --- | --- |
| Environmental Collaboration with Suppliers | 1 | 2 | 3 | 4 | 5 |
| Collaboration between product designer and suppliers | 1 | 2 | 3 | 4 | 5 |
| Supplier relationship closeness | 1 | 2 | 3 | 4 | 5 |
| The product conformance quality | 1 | 2 | 3 | 4 | 5 |
| Flexibility of suppliers | 1 | 2 | 3 | 4 | 5 |
| Green design | 1 | 2 | 3 | 4 | 5 |
| Green purchasing | 1 | 2 | 3 | 4 | 5 |
| ISO 14000 | 1 | 2 | 3 | 4 | 5 |
| Internal green production plan | 1 | 2 | 3 | 4 | 5 |
| Cleanser production | 1 | 2 | 3 | 4 | 5 |
| Degree of innovativeness of R & D green product | 1 | 2 | 3 | 4 | 5 |
| Intra-Organizational management | 1 | 2 | 3 | 4 | 5 |
| Supplier Integration | 1 | 2 | 3 | 4 | 5 |
| Eco-Design | 1 | 2 | 3 | 4 | 5 |
| Customer cooperation | 1 | 2 | 3 | 4 | 5 |
| Reverse Logistics | 1 | 2 | 3 | 4 | 5 |
| Human Technical Expertise | 1 | 2 | 3 | 4 | 5 |
| Sustainability | 1 | 2 | 3 | 4 | 5 |
| Training of suppliers and employees | 1 | 2 | 3 | 4 | 5 |
| Government regulations and standards | 1 | 2 | 3 | 4 | 5 |
| Employee involvement | 1 | 2 | 3 | 4 | 5 |
| Brand Image of Organization | 1 | 2 | 3 | 4 | 5 |
| Green awareness | 1 | 2 | 3 | 4 | 5 |
| Green Procurement | 1 | 2 | 3 | 4 | 5 |
| Green energy promotion | 1 | 2 | 3 | 4 | 5 |

| **Strongly Disagree** | **Disagree** | **Neutral** | | **Agree** | **Strongly Agree** |
| --- | --- | --- | --- | --- | --- |
| SD | DA | | NE | AG | SA |

**Environmental Performance**

|  | SD | DA | NE | AG | SA |
| --- | --- | --- | --- | --- | --- |
| Minimized the emission of hazardous substances or waste | 1 | 2 | 3 | 4 | 5 |
| Minimized the consumption of energy | 1 | 2 | 3 | 4 | 5 |
| Minimized the consumption of direct or indirect usage of material | 1 | 2 | 3 | 4 | 5 |
| Minimized the consumption of hazardous materials | 1 | 2 | 3 | 4 | 5 |
| Improved its overall environmental situation | 1 | 2 | 3 | 4 | 5 |
| Improved the compliance to environmental regulations and standards | 1 | 2 | 3 | 4 | 5 |

**Financial Performance**

|  | SD | DA | NE | AG | SA |
| --- | --- | --- | --- | --- | --- |
| Increased the market share and growth rate | 1 | 2 | 3 | 4 | 5 |
| Increased the growth in profit margin | 1 | 2 | 3 | 4 | 5 |
| Increased the level of productivity | 1 | 2 | 3 | 4 | 5 |
| Increased the growth in sales | 1 | 2 | 3 | 4 | 5 |
| Lower the cost of production or production cost per unit | 1 | 2 | 3 | 4 | 5 |
| Improved the overall customer satisfaction | 1 | 2 | 3 | 4 | 5 |

**Social Performance**

|  | SD | DA | NE | AG | SA |
| --- | --- | --- | --- | --- | --- |
| Substantially improved the overall customer retention and loyalty | 1 | 2 | 3 | 4 | 5 |
| Substantially enhanced its green image | 1 | 2 | 3 | 4 | 5 |
| Constantly paid important concern on the health and safety of the society | 1 | 2 | 3 | 4 | 5 |
| Constantly paid important concern on the society well-being in all operation | 1 | 2 | 3 | 4 | 5 |
| Constantly paid important concern on how the society response towards firm’s action | 1 | 2 | 3 | 4 | 5 |
